# Urban noise and surrounding city morphology influence green space occupancy by native birds in a Mediterranean-type South American metropolis

**DOI:** 10.1038/s41598-022-08654-7

**Published:** 2022-03-16

**Authors:** Constanza Arévalo, Juan David Amaya-Espinel, Cristián Henríquez, José Tomás Ibarra, Cristián Bonacic

**Affiliations:** 1 Fauna Australis Wildlife Laboratory, School of Agriculture and Forestry Engineering, Pontificia Universidad Católica de Chile, Avenida Vicuña Mackenna 4860, 7820436 Macul, Santiago Chile; 2 Departamento de Ecología y Territorio, Facultad de Estudios Ambientales y Rurales, Pontificia Universidad Javeriana, Cra. 7 # 40-62, Bogotá, D.C. Colombia; 3 Institute of Geography, Faculty of History, Geography and Political Science, Pontificia Universidad Católica de Chile, Avenida Vicuña Mackenna 4860, Macul, Santiago, Chile; 4 Centre for Sustainable Urban Development (CEDEUS) and Centro Interdisciplinario de Cambio Global UC, Santiago, Chile; 5 ECOS (Ecosystem-Complexity-Society) Co-Laboratory, Center for Local Development (CEDEL) and Center for Intercultural and Indigenous Research (CIIR), Villarrica Campus, Pontificia Universidad Católica de Chile, Bernardo OHiggins 501, 4930000 Villarrica, Chile; 6 Cape Horn International Center for Global Change Studies and Biocultural Conservation (CHIC) and Center of Applied Ecology and Sustainability (CAPES), Santiago, Chile; 7 School of Veterinary Medicine, Pontificia Universidad Católica de Chile, 8940000 Santiago, Chile

**Keywords:** Biodiversity, Urban ecology, Ecology, Zoology

## Abstract

Urban green spaces provide natural habitat for birds in urban landscapes, yet the effects of noise and surrounding urban morphology on bird community structure and distribution are not well understood in Latin America, the second most urbanized region in the world. Santiago of Chile is the single city belonging to the Mediterranean ecosystem in South America and is subject to extensive urbanization as seen throughout Latin America. We examined the role of 65 urban green spaces—6 large urban parks (PAR) and 59 small green spaces (SGS)—in harboring native birds during winter 2019, analyzing the quality of green areas in terms of vegetation (i.e. NDVI, native vegetation, and tree cover), exotic bird species, noise levels, and surrounding urban morphology (i.e. building height and cover). Significantly higher noise levels were detected in SGS, along with significantly greater exotic bird (n = 4) richness and abundance than PAR, which possessed significantly greater native bird (n = 25) richness and abundance. Native birds were more abundant than exotic birds in green spaces with average noise levels < 52 dB and average NDVI > 0.5. Occupancy models indicate that green space occupancy by 50% of modeled native bird species was influenced by maximum noise levels, playing a larger role than vegetation (30%) and urban morphology (0%). We stress the importance of developing networks of large green spaces in rapidly urbanizing regions, with abundant tree cover, surrounded by smaller urban morphology, and regulating noise levels to ensure the conservation of native bird communities in cities, particularly those that are threatened.

## Introduction

Over half (55%) of the world’s population resides in urban areas, a number expected to reach 68% by 2050^1^. Urban population growth has been particularly rapid in Latin America and the Caribbean, making up the world’s second most urbanized region with 81% of its population living in urban areas, and an expected 90% by 2050^1^. Urbanization poses a risk to biodiversity with the loss and fragmentation of natural habitats for countless species around the globe, while a rise in noise, light, and chemical pollution associated with urbanization further impact urban species by influencing physiological and behavioral changes^2,3^, resulting in serious challenges for biodiversity conservation.

Species can be categorized according to their tolerance to urbanization, classifying them based on their responses to urban development^4–7^. Three categories of urbanization tolerance represent a response gradient: *urban avoiders* range from extirpated in developed areas to self-sustaining in networks of natural areas within cities, *urban utilizers* range from occasionally using urban resources to breeding in developed areas, and *urban dwellers* range from having viable populations in both natural and urban areas to being dependent on urban areas for their survival^5^.

As the global population becomes increasingly more urban, we can expect a rise in anthropogenic noise within the world’s soundscapes. Sound serves as an important stimulus for numerous species, carrying large amounts of information used in intra- and inter-species communication^8^. When noise becomes permanent in a system, as occurs with urbanization, communication among individuals becomes limited, altering their ability to forage, hide from predators, and reproduce^9–11^.

Certain animal species, such as songbirds, are particularly susceptible to changes in the soundscape^10,12^, with studies attributing reduced reproductive success, bird fitness, bird densities, bird community richness and diversity, as well as changes to species interactions in urban areas to anthropogenic noise^10,13,14^. Birds may be excluded on a purely acoustic basis from otherwise suitable habitats^12,15^, with the anthrophony (human-produced sounds) acting as a source of habitat degradation and fragmentation that affects the distribution of bird communities. This acoustic fragmentation, responsible for developing song differences between urban and non-urban birds of the same species^16–20^, may result in reproductive isolation^21^.

Amid the negative impacts of urbanization on bird communities, urban green spaces provide pockets of habitat for birds. Studies conducted in urban parks have found that bird species richness is positively related to the area of green spaces and negatively related to anthropogenic noise levels^22–24^. As residential density increases with a growing global population, urban parks are predicted to lose their capacity to provide habitat for urban wildlife^23^. A series of ecological conditions that occur in city green spaces influence bird richness and abundance. First, road coverage and building density. Second, the presence of invasive species and urban-dweller generalist bird species, and third, anthropogenic noise levels^25^. Particularly, the effects of anthropogenic noise on bird communities have been suggested to go beyond the community level, altering bird species richness and composition at a regional scale^22,26^.

Due to the significance of urban green spaces for the survival and reproduction of bird communities within cities, it is imperative that the often-unnoticed effects of urban morphology surrounding these sites and the noise within them be studied to better understand how they affect bird community structure and distribution. Latin America, as one of the fastest urbanizing regions in the world and the most diverse in avian species, is lacking in such studies^27^, with most studies on wildlife and anthropogenic noise being conducted in North America and Europe^28^, and most research on avian communities in Latin American urban areas generally conducted in the most populated countries^27^. Furthermore, historically, most research on urban birds has looked at the influences of vegetation, with urban noise and impervious surface cover gaining traction in recent years^27^, but little is known about the combined influences of those variables with urban morphology on bird communities in Latin American cities. Santiago of Chile is one of the large metropolises of Latin America and stands out for its high-density value in terms of inhabitants per hectare, along with cities like Bogotá, Caracas, São Paulo, and Mexico City^29^. With 88.4% of its population currently residing in urban areas^30^, Chile is one of the South American countries with the highest urban population concentrated in the single Mediterranean-type ecosystem of South America. Even though birds are the taxa with the least number of endemic species relative to other terrestrial vertebrates in Chile, they include unique species like the Chilean mockingbird (*Mimus thenca*), moustached turca (*Pteroptochos megapodius*), slender-billed parakeet (*Enicognathus leptorhynchus*), among others whose conservation status has been affected by intensive agriculture and urbanization.

This study aimed to understand the influence of urbanization on native bird species richness, abundance, and green space occupancy in an exemplary South American metropolis. Specifically, we sought to determine how native bird richness and abundance in urban green spaces are influenced by anthropogenic noise, the surrounding city morphology, exotic bird species, and vegetation in green spaces. We further used occupancy modeling techniques to predict native urban avoider, urban utilizer, and urban dweller bird species presence in urban green spaces, given noise levels, surrounding building height and cover, and vegetation variables. We expected: (i) native bird richness and abundance to decrease with rising noise levels, building height, building cover, and exotic bird abundance; (ii) probability of green space occupancy by native birds to decrease as noise levels, building height, and building cover increase; and (iii) native urban avoider species to show the most sensitivity to noise and urban morphology, and thus show increased occupancy in large urban parks, and native urban dwellers to be the most tolerant to urbanization and be more commonly found in small green spaces.

## Results

We detected 29 bird species (Appendix S2) (86% native [n = 25] to Chile; 14% exotic [n = 4]) within the green spaces of Santiago, all categorized as being of ‘least concern’ in the International Union for Conservation of Nature (IUCN) Red List. Generally, native bird richness and abundance were greatest in large urban parks (PAR; area > 10 ha), while exotic bird abundance was greater in small green spaces (SGS; area < 10 ha) (“Appendix S1”). Native bird richness and abundance decreased with increasing noise levels and building height surrounding green spaces, while increasing with vegetation cover (“Appendix S3”). Exotic bird abundance increased with rising noise levels and building height, decreased with vegetation cover, and exotic bird richness decreased with tree cover (“Appendix S3”). Native birds reached lesser abundances than exotic birds when average noise levels reached over 52 dB, and, once vegetation cover in green spaces reached an average NDVI value of 0.5 or more, native birds tended to reach higher abundances than exotic birds (“Appendix S4”).

### Native bird tolerances to urbanization conditions

**Figure 1:**
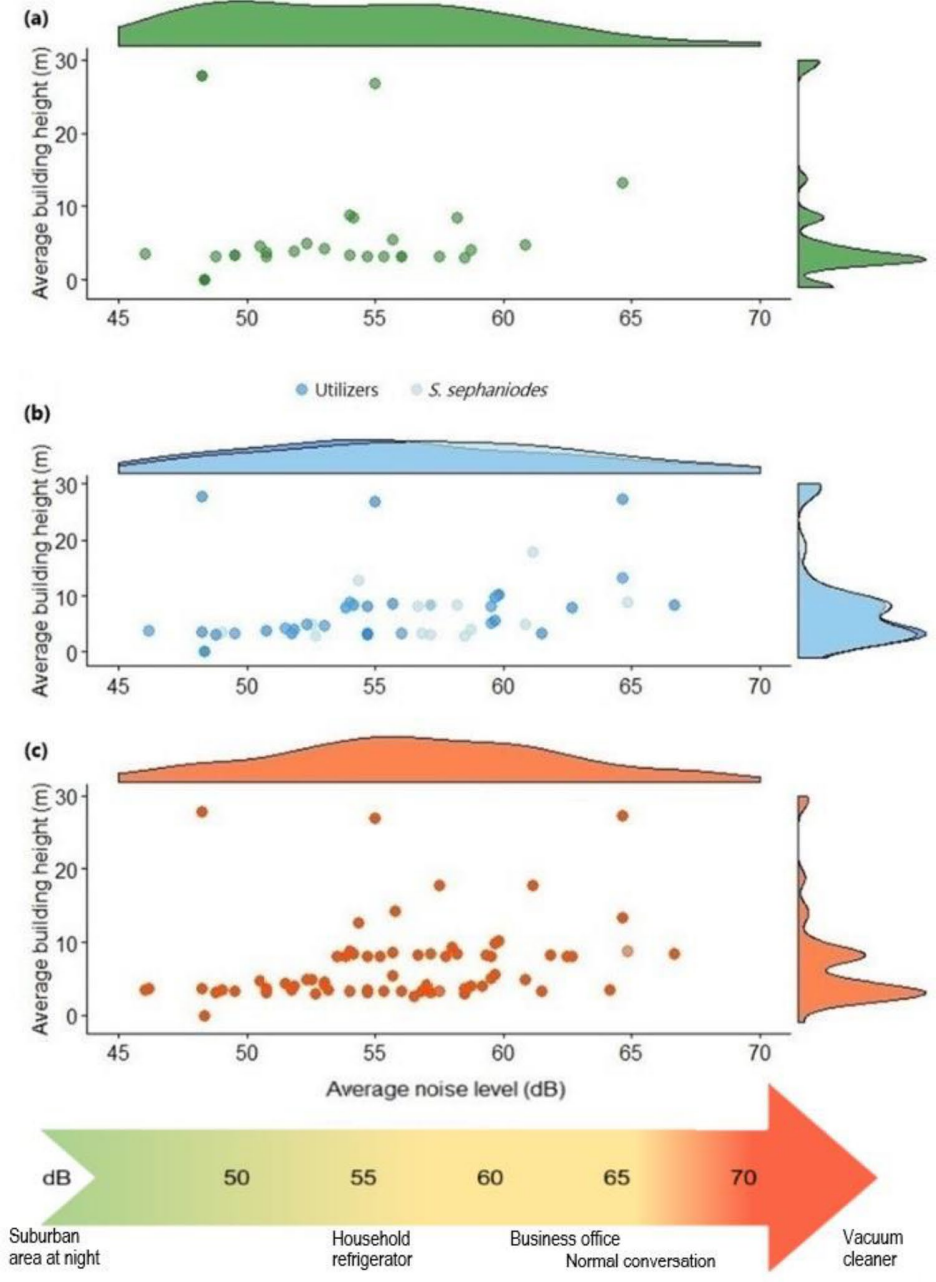
Average noise levels in green spaces and average building height surrounding green spaces at which native bird species occur: a) urban avoiders, b) urban utilizers, with *Sephanoides sephaniodes* considered separately, and c) urban dwellers. Marginal density plots indicate the distribution of native birds at varying average noise levels in green spaces (top) and varying average building height surrounding green spaces (right). Decibel level comparisons (based on values established by Yale EHS) are displayed in the arrow at the bottom for reference.

Average noise levels in green spaces shared significant correlations with the richness and abundance of native urban avoider (n = 7), urban utilizer (n = 8), and urban dweller (n = 12) species, indicating an important negative trend in the richness and abundance of all native bird species as average noise levels rose in green spaces (Table 1). Additionally, a significant positive correlation was found between NDVI and urban utilizer abundance in green spaces, a negative relation between native vegetation in green spaces and native urban utilizer richness and abundance, as well as a significant negative relation between average building height surrounding green spaces and urban avoider and urban dweller species richness and abundance. Native urban avoider species displayed the least tolerance to anthropogenic noise and building height surrounding green spaces, rarely detected in green spaces with average noise levels over 60 dB and average building height over 10 m, with most found in green spaces with average noise levels of 50–55 dB and surrounded by buildings 4–6 m tall (Fig. 1a). Unlike most native urban utilizer species, the green-backed firecrown (*Sephanoides sephaniodes*) was found in most green spaces, for which reason it was considered separately from other urban utilizers (Fig. 1b). The green-backed firecrown’s tolerance to anthropogenic noise was more like that of urban dwellers, found throughout most noise levels, though more commonly in green spaces with average noise levels between 50 and 65 dB (Fig. 1c), while other urban utilizers were most detected in green spaces with average noise levels around 55 dB, decreasing rapidly with noise levels higher than that (Fig. 1b). All native birds showed sensitivity to building height, with few native birds detected in green spaces surrounded by buildings more than 10 m tall on average. Meanwhile, there was not sufficient evidence that building cover surrounding green spaces, nor tree cover in green spaces, share significant relations with native urban avoider, urban utilizer, and urban dweller richness and abundance in green spaces (Table 1).

**Table 1:** Pearson/Spearman correlation coefficients and associated p-values for native urban avoider, urban utilizer, and urban dweller groups in green spaces of Santiago, Chile.

	Richness			Abundance		
	Urban avoiders	Urban utilizers	Urban dwellers	Urban avoiders	Urban utilizers	Urban dwellers
Average noise	− 0.37 *p* = 0.002*	− 0.29 *p* = 0.016*	− 0.24 *p* = 0.049*	− 0.35 *p* = 0.003*	− 0.31 *p* = 0.009*	− 0.36 *p* = 0.003*
NDVI	− 0.10 *p* = 0.399	0.17 *p* = 0.172	0.01 *p* = 0.904	− 0.07 *p* = 0.568	0.38 *p* = 0.001*	− 0.03 *p* = 0.783
Tree cover	− 0.06 *p* = 0.601	− 0.14 *p* = 0.256	0.02 *p* = 0.861	− 0.02 *p* = 0.860	0.08 *p* = 0.530	− 0.09 *p* = 0.483
Native vegetation	− 0.05 *p* = 0.695	− 0.25 *p* = 0.041*	− 0.01 *p* = 0.904	− 0.03 *p* = 0.834	− 0.27 *p* = 0.028*	− 0.13 *p* = 0.270
Average building height	− 0.25 *p* = 0.040*	0.02 *p* = 0.863	− 0.24 *p* = 0.049*	− 0.25 *p* = 0.036*	− 0.20 *p* = 0.095	− 0.35 *p* = 0.003*
Proportion building cover	0.02 *p* = 0.872	− 0.18 *p* = 0.149	0.02 *p* = 0.873	0.01 *p* = 0.954	− 0.09 *p* = 0.460	− 0.05 *p* = 0.675

*Significant correlation, given significance level α = 0.05.

Generally, noise levels were found to be greatest in green spaces surrounded by taller urban morphology, while there was not enough evidence indicating that building cover shared a relation with noise.

### Detection and occupancy probability models

Detectability remained constant across surveys and surveyed sites for the Chilean mockingbird (*Mimus thenca*), the only endemic bird species detected in the study area, not affected by any one variable. Detectability varied across survey sites for the remaining 13 native bird species, changing with the maximum noise level recorded during surveys (14%), vegetation cover (29%), tree cover (21%), native vegetation (14%), building cover surrounding green spaces (7%), and with a combination of average building height and maximum noise level (7%). Detectability decreased with rising noise levels, native vegetation, building height, and building cover surrounding green spaces, while increasing with vegetation cover.

**Figure 2:**
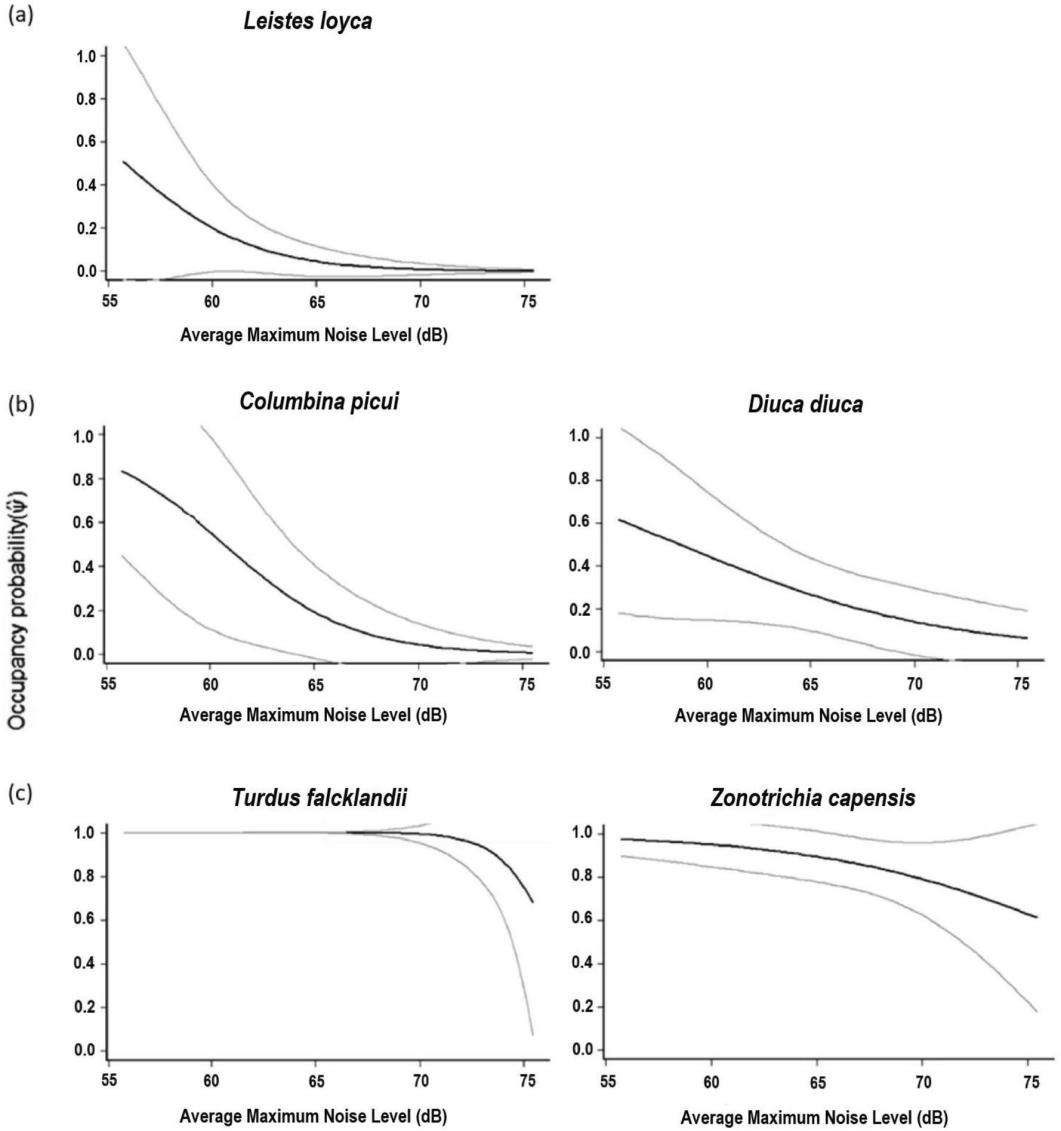
Occupancy probability plots for species whose occupancy was most influenced by average maximum noise levels in green spaces in Santiago, Chile: (**a**) native urban avoider species, (**b**) native urban utilizer species, and (**c**) native urban dweller species. Plots are based on the models summarized in Table 2. Gray lines represent 95% confidence intervals.

Green space occupancy was most influenced by the maximum noise levels recorded in green spaces for 50% of modeled native bird species (Table 2), with urban avoider and urban utilizer species showing the lowest noise tolerances, and exotic species displaying the highest tolerances to noise, with some species present especially in high-noise green spaces (the rock pigeon (*Columba livia*) and the monk parakeet (*Myiopsitta monachus*)). The picui ground dove (*Columbina picui*) and long-tailed meadowlark (*Leistes loyca*) were the modeled native species most sensitive to noise, their occupancy probabilities decreasing rapidly as the average maximum noise level of a green space reached 55–65 dB, approaching an occupancy probability of 0 when average maximum noise levels reached over 65 dB (Fig. 2a, b). The common diuca finch (*Diuca diuca*) and rufous-collared sparrow (*Zonotrichia capensis*) were also quite sensitive to noise, their occupancy probabilities decreasing as average maximum noise levels reached above 55 dB (Fig. 2b, c), but the decrease in their occupancy probabilities was more gradual than for the other two species. The austral thrush (*Turdus falcklandii*) displayed the least sensitivity to noise, present in all green spaces with an average maximum noise level below approximately 73 dB (Fig. 2c). Following maximum noise level as the most influential covariate in occupancy probability, tree cover in green spaces was most influential for 20% of native birds, green space occupancy remained constant across sites for 20% of native birds, and, for the remaining 10%, occupancy was influenced by vegetation cover (Table 2).

**Table 2:** Best occupancy detection probability models for bird species detected in Santiago, Chile, showing estimated detection (*p*) and occupancy (Ψ) probabilities.

Urbanization tolerance	Common name^a^	Scientific name	Model^b^	Est. *p*	Naïve ψ	Est. ψ
Avoider	Long-tailed meadowlark	*Leistes loyca*	ψ(MNA) p(NDVI)	0.565	0.073	0.048
Utilizer	Picui ground dove	*Columbina picui*	ψ(MNA) p(NV)	0.105	0.130	0.199
Utilizer	Common diuca finch	*Diuca diuca*	ψ(MNA) p(Tree)	0.249	0.203	0.374
Utilizer	Chilean mockingbird	*Mimus thenca*	ψ(NDVI) p(.)	0.456	0.275	0.327
Utilizer	Green-backed firecrown	*Sephanoides sephaniodes*	ψ(Tree) p(NDVI)	0.909	0.913	0.970
Utilizer	Black-chinned siskin	*Spinus barbatus*	ψ(.)p(MN)	0.116	0.072	0.218
Utilizer	Southern lapwing	*Vanellus chilensis*	ψ(Tree) p(NDVI)	0.196	0.145	0.281
Urban dweller	Feral Pigeon*	*Columba livia*	ψ(MNA + H) p(NDVI)	0.933	0.884	0.952
Urban dweller	Chimango caracara	*Milvago chimango*	ψ(.) p(MN)	0.420	0.507	0.658
Urban dweller	Shiny cowbird*	*Molothrus bonariensis*	ψ(MNA) p(Tree)	0.457	0.652	0.883
Urban dweller	Monk parakeet*	*Myiopsitta monachus*	Ψ(NV) p(D)	0.728	0.812	0.879
Urban dweller	House sparrow*	*Passer domesticus*	ψ(NDVI) p(NDVI)	0.439	0.449	0.482
Urban dweller	Austral thrush	*Turdus falcklandii*	ψ(MNA) p(D)	0.991	0.986	0.999
Urban dweller	Rufous-collared sparrow	*Zonotrichia capensis*	ψ(MNA) p(NV)	0.621	0.797	0.895

^a^Exotic species marked with *.

^b^Variables: maximum noise level (MN), average maximum noise level (MNA), vegetation cover (NDVI), proportion native vegetation (NV), proportion tree cover (Tree), average building height surrounding green space (H), building cover surrounding green space (D), constant occupancy/detection across sites (.).

## Discussion

Our research determined noise to share a potentially important negative relationship with native bird richness and abundance and appears to be the most limiting factor in green space occupancy by native bird species, more so than the type and amount of vegetation present in urban green spaces, and more so than urbanization itself, represented as building height and cover surrounding green spaces. Thus, noise is potentially acting as an invisible source of habitat degradation, limiting the bird species capable of inhabiting an area, regardless of whether the appropriate vegetative conditions exist.

As predicted, native urban avoiders reached their maximum abundances in PAR, which, given their high vegetation cover and large size, act as patches of natural habitat in cities. Native urban utilizers tended to be found in more suburban areas, and urban dwellers, both native and exotic, were detected in green spaces of all noise levels. All exotic bird species were urban dwellers, referring to their high tolerance to urbanization^5,25^, thus reaching the high abundances observed, particularly in SGS.

SGS possessed higher average noise levels and greater exotic bird abundance than PAR, which presented significantly higher numbers of native bird richness and abundance. The potential influence of noise on native bird species first becomes evident when we consider that native bird abundance tended to rise above the generally high abundance of exotic birds when average noise levels in green spaces reached below 52 dB (it should be noted that, according to the Chilean Noise Norm No. 146, the maximum allowable noise levels generated by *fixed* sources in residential areas of Santiago is 55 dB during the day, 7 a.m.–9 p.m.). The negative relations between noise and urban avoider, urban utilizer, and urban dweller species richness and abundance further indicate how noise may be regulating the native bird species present in green spaces, affecting urban avoider richness the most and urban dweller richness the least, while influencing the abundance of all native bird species rather similarly. Meanwhile, building height surrounding green spaces negatively influenced native urban avoider and urban dweller richness and abundance, with the greatest influence on urban dweller abundance, yet all native birds were less likely to be detected in green spaces surrounded by buildings over 10 m tall on average.

The importance of vegetation for native bird communities also cannot be denied, given that native birds reached higher abundances than exotic birds when vegetation cover reached an average NDVI value greater than 0.5. Results from this study thus suggest that exotic birds begin to replace native birds in terms of abundance as noise levels rise in urban green spaces, vegetation cover decreases, and building height surrounding green spaces increases, with native urban avoider species being the least tolerant to the influences of urbanization, and, consequently, the first to disappear when noise levels and building height become too great. The observed negative relationship between native bird species richness and maximum noise levels, and the positive relationship with vegetation cover, are comparable to results seen in other Neotropical cities^24,26^, yet our results indicate that the relationships between these variables and bird abundance are stronger. This may indicate how bird abundance fluctuates in green spaces as some birds temporarily leave during noisy events or become quieter and more cryptic under noisy conditions^26^, while noise also negatively influences bird species richness by filtering the species that can inhabit areas of varying noise levels.

Detection probability models found native bird detectability to mostly increase with vegetation cover and tree cover in urban green spaces, except for the common diuca finch, whose detectability decreased with rising tree cover. Some of the bird species that displayed the lowest detection probabilities, such as the picui ground dove and fire-eyed diucon (*Xolmis pyrope*), are not frequently found in cities and possess vocalizations that are unlikely to be heard well in high-noise areas due to their low frequencies, making them more easily masked by the anthrophony, characterized by its low frequency and high intensity^31^. Consequently, birds whose vocalizations are similar in frequency and amplitude to the anthrophony were more commonly or exclusively found in green spaces that registered low noise levels, their detectability also decreasing with rising noise, as was the case with the fire-eyed diucon.

Urban green space occupancy by native bird species was mainly influenced by average maximum noise levels recorded in green spaces. Of the modeled native species, the long-tailed meadowlark and the picui ground dove, an urban avoider and an urban utilizer species respectively, were the species most sensitive to noise, their probability of occupying green spaces with average maximum noise levels over 55 dB decreasing rapidly and approaching zero when over 65 dB. Meanwhile, the austral thrush, an urban dweller species, was by far the most tolerant to noise of the native birds, its presence probability just beginning to decrease when average maximum noise levels reached over 73 dB in green spaces. The differing tendencies of urban avoiders, urban utilizers, and urban dwellers to occupy green spaces of varying noise levels is thus evident, with native urban dweller species more likely to occupy higher noise urban green spaces than urban avoiders and utilizers, seemingly more adapted to the high noise levels that come with inhabiting a busy city. Nonetheless, although native urban dwellers displayed greater noise tolerances than urban avoiders and utilizers, their presence in city parks can also be expected to diminish if noise levels become too high, which for the most tolerant of the native birds, means reaching an average maximum level of 73 dB or more, but 55 dB or more for less tolerant species.

No relation was found between vegetation cover and noise, and some of the highest noise levels were recorded in PAR. This suggests that PAR, often considered to be quiet and peaceful areas to escape the busyness of city life, can reach noise levels as high as those recorded in SGS, reducing the quality of the greatest sources of natural habitat for birds and other wildlife in cities.

The results from this study regarding the influence of noise on bird communities support previous studies indicating that birds may be excluded from suitable habitats on account of the acoustic conditions of the local environment^12,15^. Despite abundant vegetation in PAR and some SGS, certain bird species, particularly urban avoiders and utilizers, were less likely to occupy areas that presented high noise levels. However, it is important to consider other potential influencing factors, such as predators (e.g., dogs and cats) and food availability, both of which could be linked to pedestrians and could therefore also increase noise levels in green spaces. Furthermore, in an effort to focus on the influence of anthropogenic variables on urban birds (i.e., urban morphology, noise, and vegetation type and cover), this study did not consider the size of urban green spaces as a variable in occupancy modeling, but as the results of this study suggest and others in Latin America have shown^23,32^, green space size is likely an influencing factor that should be considered in future studies. Another variable worth considering would be road coverage, which undoubtedly plays a role in noise levels, particularly for SGS.

Measures to control the COVID-19 pandemic have significantly reduced noise levels in major cities worldwide^33–35^. Noise reduction in the San Francisco Bay Area, characterized by a Mediterranean climate like Santiago, resulted in songbirds rapidly occupying newly available acoustic niches within urban soundscapes and maximizing communication through higher performance songs^35^. Consequently, native bird species not commonly found in high-noise areas, mainly urban avoider and utilizer species, may now be found in greater abundance at the community level in urban green spaces where they had been scarce or non-existent during this study, conducted pre-pandemic. Furthermore, if average noise levels dropped below 52 dB in Santiago green spaces due to region-wide shut-down measures, native birds may reach higher abundances than exotic birds. The negative effects of urban noise on bird communities are extensive, yet recent research indicating birds’ rapid adaptability and improved vocal performance when noise levels are significantly lowered provides hope. Native bird species susceptible to noise may stand a chance despite growing urbanization, if noise levels in urban green spaces are regulated.

Rapid urban expansion in Latin America places natural ecosystems at great risk, reducing or altogether eliminating natural habitats for native birds and other wildlife, making urban green spaces necessary for their persistence, especially in biodiversity hotspots like central Chile. As this study illustrates, noise associated with urbanization plays a significant role in influencing green space occupancy by native bird species, and, quite possibly, other animal species dependent on acoustic signaling (e.g., amphibians and mammals). Given the recreational role of urban green spaces in cities, noise regulation within these areas should be considered, while also considering how city morphology may impact bird communities. This study exemplifies how, in addition to noise, the size of urban green spaces and the vegetation cover in them, particularly tree cover, are vital aspects to consider in city planning in order to preserve native bird communities in urban systems. Large urban parks held significantly richer bird communities than small green spaces, with greater native bird richness and abundance. Therefore, it is imperative that science and city planning collaborate to develop cities with networks of large green spaces with abundant tree cover, surrounded by smaller urban morphology, where noise is regulated and maintained at tolerable levels for native birds. There is a clear need to move towards biophilic city planning to harmonize urban growth and the protection and expansion of networks of green areas that generate habitat for birds that, in turn, provide important ecosystem services to cities.

## Methods

### Bird surveys and noise measurements

**Figure 3:**
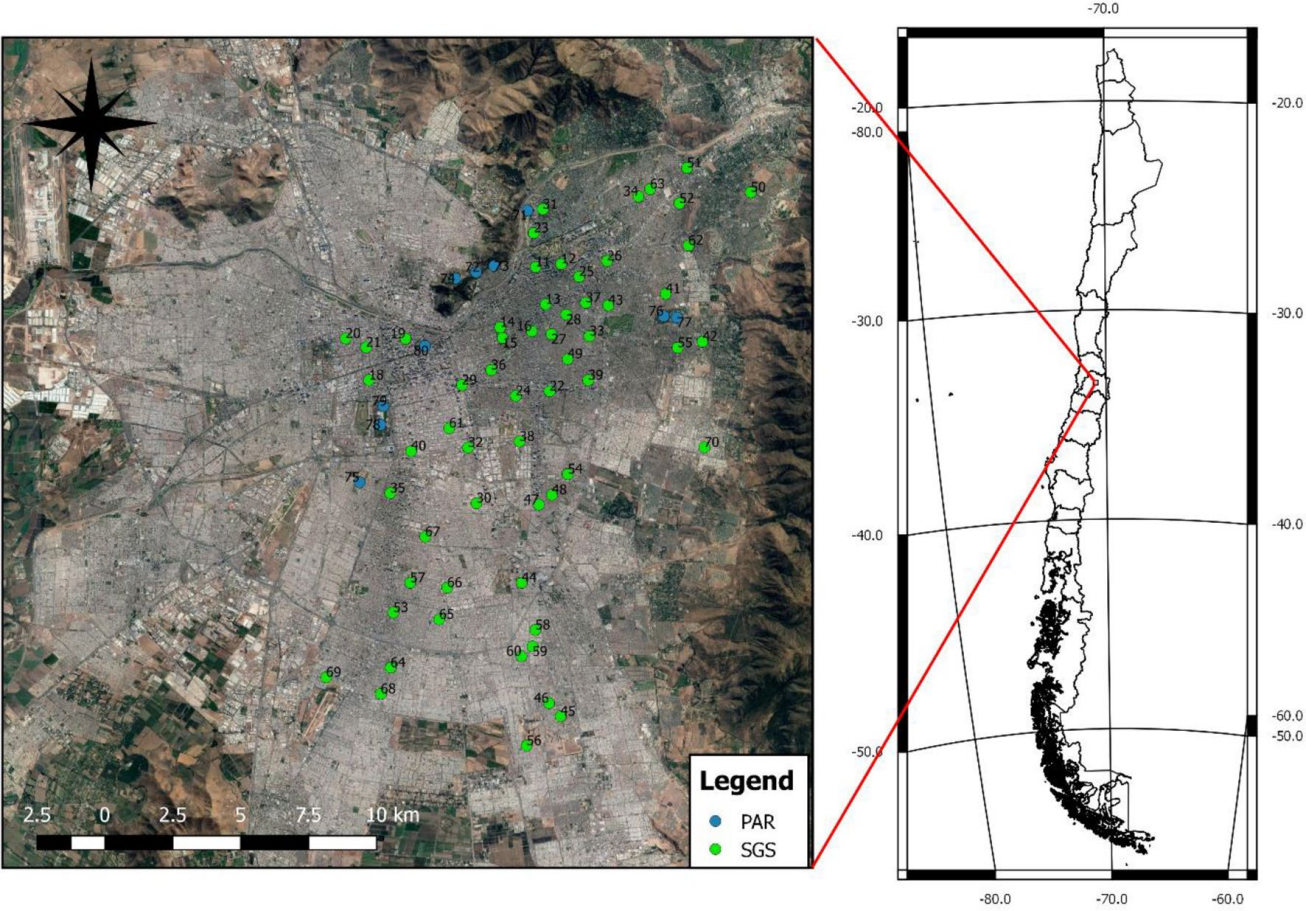
Study area within the Metropolitan Region of central Chile. Green points represent small green spaces (SGS) and blue points are large urban parks (PAR) sampled during winter 2019. Map was created using QGIS version 2.18.23 (https://www.qgis.org) with the Google Satellite plugin for QGIS (Map data^©^ 2015 Google).

We conducted this study in 59 small green spaces (SGS) and 6 large urban parks (PAR) in the Metropolitan Region of Santiago (MRS) in central Chile (Fig. 3). Located between the Andes mountains and a coastal mountain range, the MRS possesses a semi-arid Mediterranean climate, with hot, dry summers and cold, wet winters^36^. Due to its highly endemic vegetation, the Mediterranean region of central Chile is considered a biodiversity hotspot, with sclerophyllous shrubland and woodland making up the dominant vegetation^37^. The region holds about 40% of Chile’s population, of which 96.3% resides in urban areas^30^. SGS, also known as “pocket parks” or “plazas”, ranged in size from 0.5 to 2 ha and were independent of one another, separated by a distance of at least 250 m from other surveyed SGS and PAR^25^. We sampled 6 PAR of area > 10 ha as control points, with 1–3 survey points in each (generally located in the center of the park), depending on park size, for a total of 10 PAR sample sites, ensuring a distance of at least 250 m between survey points. Green spaces were identified using a digital layer of green area developed for the MRS at a scale of 1:5000, updated using Landsat 8 OLI Thematic Mapper satellite imagery from November 2013^25^.

We conducted point count surveys at all SGS and PAR once a month during winter 2019 (May–August), for a total of three surveys per green space. To capture the morning chorus, the point of maximum biophony activity^38^, we conducted bird surveys within four hours from sunrise. We altered the order of bird surveys each month so as to never sample a green space at the same time, to account for varying noise levels throughout the morning, which in turn could alter the bird species present during a survey^26^. We used a radius of 50 m, recording all bird species detected visually and acoustically during a 10-min period, along with the number of individuals of each species. Additionally, we determined noise levels at the beginning and end of each bird survey for a period of 30 s using the NIOSH Sound Level Meter app for smartphones combined with the Dayton Audio iMM-6 calibrated measurement microphone, measuring average continuous equivalent sound pressure levels (LA_eq_) and maximum equivalent sound pressure levels (L_max_) using slow A frequency weighting.

### Urban matrix measurements

As a measure of the urban matrix resulting from urbanization, we considered average building height and building cover within a circular buffer of 200 m from the central point of each urban green space, obtained in 2015^25^. We calculated the proportion of building cover within the buffer using the Normalized Difference Vegetation Index (NDVI) to map the percentage of the buffer covered by vegetation and took the difference. Average building height was calculated using a raster layer with cadastral information for the study area. Building height was validated through Google Street View 3D models, estimating a height of 2.70 m per built floor. We determined average building height surrounding urban green spaces for a random sample (n = 15) of SGS in 2020 using the same method, but no significant differences were found between 2015 and 2020 urban morphology data (*t* test *p* value = 0.922).

### Vegetation cover and composition

We determined the average NDVI for each urban green space as an estimate of vegetation cover, using QGIS 2.18.23 to process Landsat 8 OLI Thematic Mapper satellite imagery of the study area taken in September 2019, with values close to zero indicating bare soil or urban areas, and values closer to 1 implying dense vegetation.

Additionally, we estimated the proportion of native vegetation and tree cover (vegetation > 2 m tall) in each green space, following 40 points along the border of 50 × 50 m plots at the center of each survey site (every 2.5 m along 4 transects of 50 m), using frequency as a measure of cover^25^.

### Statistical analysis

We considered richness to be the total number of bird species detected per urban green space during the study period and abundance to be the average number of individuals of each species detected per urban green space throughout the point counts. LA_eq_ measurements were averaged to obtain the average noise level per green space (NW), and the same was done with L_max_ measurements, giving an average maximum noise level per green space (MNA). We tested all site variables (NW, MNA, average building height, building cover, NDVI, proportion native vegetation, and tree cover) for collinearity. In the event of high correlation (*r* > 0.7), only one variable was considered, as was the case with NW and MNA. Correlation analyses were also run between site variables and species richness and abundance.

We modeled native and exotic bird abundance in relation to site variables with generalized linear models (GLMs) using R version 3.6.0 and the R package *ggplot2*. To visualize the distribution of native birds under the influence of the most significant urban variables, we created scatterplots with marginal density plots for each urban tolerance category (urban avoiders, urban utilizers, urban dwellers) considering the urbanization variables that had the highest correlations with native bird richness and abundance. Species urban tolerances were determined following the classification method proposed by Fischer et al. (2015), considering the spatial and temporal frequency of species observations^25^.

### Detection and occupancy modeling

We developed occupancy models using the R package *unmarked*^39^ to determine the true presence and absence probabilities of bird species at sampling sites. The probability of detecting a species and its probability of occupying a green space both follow a Bernoulli model to which we incorporated data from repeated surveys, as well as site (i.e. MNA, average building height surrounding green spaces [H], proportion of building cover surrounding green spaces [D], vegetation cover [NDVI], proportion of tree cover [Tree], and proportion of native vegetation in green spaces [NV]) and observation (i.e. maximum noise level during point count [MN]) covariates to test how these variables influence species detection and green space occupancy.

We determined the best detection probability model for each bird species detected, keeping occupancy (ψ) constant across all sites and considering all observation and site covariates. We used a predetermined set of models (“Appendix S5”), which included individual covariates as well as combinations of urban morphology and noise covariates. Models were selected based on Akaike Information Criteria (AIC), where only models with AIC ≤ 2.0 were considered in the model selection process. The model with the lowest AIC value and greatest Akaike weight (w) was selected as the best model for each bird species.

The best detectability model for each bird species was then incorporated into the occupancy model selection process, where we tested all site covariates to obtain the best occupancy model. Model selection was carried out in the same manner as with detectability, using AIC to determine the best model for each bird species. Species that displayed estimated detection probabilities (*p*) < 0.1 or that displayed a broad confidence interval associated with their occupancy probability (ψ) were not included due to the uncertainty associated with the expected detection of those species and/or green space occupancy by those species. 15 native bird species, mainly urban avoiders and urban utilizers, were discarded from occupancy modeling due to low detection rates. However, species eliminated from occupancy modeling resulting from wide ψ confidence intervals, but that presented *p* > 0.1 and an appropriate associated confidence interval, were considered when determining the most influential covariates on species detectability. We created occupancy models for 10 native bird species (Table 2; Fig. 2) and 4 exotic bird species (Table 2). Covariates influencing detectability were considered for 14 native bird species (the 10 native species in Table 2 and *Anairetes parulus, Curaeus curaeus, Troglodytes aedon, and Zenaida auriculata*, which displayed appropriate p and associated confidence intervals, but inflated Ψ).

## Supplementary Information

Supplementary Information.

## Data Availability

The datasets generated and analyzed during the current study, along with the R code generated to analyze the data, are available from the corresponding author upon reasonable request.
