# Supplementary material for: Urban noise and surrounding city morphology influence green space occupancy by native birds in a Mediterranean-type South American metropolis
Source: Sci Rep. 2022 Mar 16;12:4471. doi: 10.1038/s41598-022-08654-7 (PMC8924563; doi:10.1038/s41598-022-08654-7)
Supplement: Supplementary file 1 — Supplementary Information. [file 41598_2022_8654_MOESM1_ESM.pdf]

Urban noise and surrounding city morphology influence green space occupancy by native birds in a Mediterranean-type South American metropolis

Supplementary Information

Appendix S1 Mean (SD) values for bird richness, abundance, noise level variables, and vegetation variables in small green spaces (SGS) and large urban parks (PAR) in the Metropolitan Region of Santiago during winter 2019.

	SGS	PAR	t-test p-value[a]
Total bird richness	10.102 (2.398)	10.5 (3.536)	0.738
Proportion native bird species	0.696 (0.095)	0.821 (0.113)	0.007*
Proportion exotic bird species	0.304 (0.095)	0.179 (0.113)	0.007*
Total bird abundance	52.237 (21.752)	40.2 (17.731)	0.076
Native bird abundance	18.169 (6.846)	28.9 (9.792)	0.007*
Exotic bird abundance	34.068 (24.388)	11.3 (12.311)	0.000*
NW[b] (dB)	56.501 (4.950)	53.650 (3.879)	0.058
MNA[c] (dB)	65.359 (4.534)	62.150 (4.141)	0.044*
NDVI[d]	0.376 (0.057)	0.521 (0.098)	0.001*
Proportion native vegetation	0.098 (0.122)	0.183 (0.241)	0.302
Proportion tree cover	0.514 (0.157)	0.558 (0.261)	0.620

[a] Values marked with * indicate significant differences between SGS and PAR with 95% confidence
[b] Average noise level in green spaces
[c] Average maximum noise level in green spaces
[d] Average Normalized Difference Vegetation Index

Appendix S2 List of bird species registered in 59 small green spaces (SGS) and 6 large urban parks (PAR) in the Metropolitan Region of Santiago, Chile. Urbanization tolerances were determined by Amaya-Espinel et al. (2019).

Scientific Name	Common Name	Family	Origin	Urbanization Tolerance	Avg. Abundance SGS	Avg. Abundance PAR
Anairetes parulus	Tufted Tit-Tyrant	Tyrannidae	Native	Urban Dweller	0.752	1.083
Colorhamphus parvirostris	Patagonian Tyrant	Tyrannidae	Native	Urban Avoider	0.000	0.542
Columba livia	Feral Pigeon	Columbidae	Exotic	Urban Dweller	20.303	2.167
Columbina picui	Picui Ground Dove	Columbidae	Native	Urban Utilizer	0.218	0.083
Curaeus curaeus	Austral Blackbird	Icteridae	Native	Urban Dweller	0.279	1.458
Diuca diuca	Common Diuca-Finch	Thraupidae	Native	Urban Utilizer	0.255	0.458
Elaenia albiceps	White-crested Elaenia	Tyrannidae	Native	Urban Utilizer	0.012	0.000
Falco sparverius	American Kestrel	Falconidae	Native	Urban Avoider	0.000	0.083
Geranoaetus polyosoma	Variable Hawk	Accipitridae	Native	NA	0.000	0.042
Larus dominicanus	Kelp Gull	Laridae	Native	NA	0.030	0.000
Leptasthenura aegithaloides	Plain-mantled Tit-Spinetail	Furnariidae	Native	Urban Dweller	0.164	0.458
Milvago chimango	Chimango Caracara	Falconidae	Native	Urban Dweller	0.394	4.083
Mimus thenca	Chilean Mockingbird	Mimidae	Native	Urban Utilizer	0.376	0.375
Molothrus bonariensis	Shiny Cowbird	Icteridae	Exotic	Urban Dweller	3.461	4.042
Myiopsitta monachus	Monk Parakeet	Psittacidae	Exotic	Urban Dweller	9.394	6.667
Parabuteo unicinctus	Harris's Hawk	Accipitridae	Native	Urban Avoider	0.006	0.000

Passer domesticus	House Sparrow	Passeridae	Exotic	Urban Dweller	1.345	0.542
Phytotoma rara	Rufous-tailed Plantcutter	Cotingidae	Native	Urban Utilizer	0.121	0.083
Sephanoides sephaniodes	Green-backed Firecrown	Trochilidae	Native	Urban Utilizer	2.352	4.167
Spinus barbatus	Black-chinned Siskin	Fringillidae	Native	Urban Utilizer	0.133	0.000
Sturnella loyca	Long-tailed Meadowlark	Icteridae	Native	Urban Avoider	0.024	0.792
Tachycineta meyeni	Chilean Swallow	Hirundinidae	Native	Urban Avoider	0.152	0.125
Troglodytes aedon	House Wren	Troglodytidae	Native	Urban Dweller	0.491	0.583
Turdus falcklandii	Austral Thrush	Turdidae	Native	Urban Dweller	7.848	7.917
Vanellus chilensis	Southern Lapwing	Charadriidae	Native	Urban Utilizer	0.121	3.167
Veniliornis lignarius	Striped Woodpecker	Picidae	Native	Urban Avoider	0.018	0.042
Xolmis pyrope	Fire-eyed Diucon	Tyrannidae	Native	Urban Avoider	0.018	0.333
Zenaida auriculata	Eared Dove	Columbidae	Native	Urban Dweller	2.715	2.542
Zonotrichia capensis	Rufous-collared Sparrow	Passerellidae	Native	Urban Dweller	1.212	1.042

Appendix S3 Spearman correlation coefficients and associated p-values for native and exotic bird species in green spaces of Santiago, Chile.

	Native		Exotic	
	Richness	Abundance	Richness	Abundance
Average noise	-0.41<br>p = 0.0004*	-0.45<br>p = 0.0001*	0.00<br>p = 0.983	0.33<br>p = 0.006*
Building height	-0.2<br>p = 0.104	-0.39<br>p = 0.001*	-0.03<br>p = 0.828	0.27<br>p = 0.027*
Building cover	-0.09<br>p = 0.461	-0.11<br>p = 0.381	-0.01<br>p = 0.916	0.18<br>p = 0.130
NDVI	0.01<br>p = 0.961	0.16<br>p = 0.197	-0.13<br>p = 0.276	-0.29<br>p = 0.015*
Native vegetation	-0.13<br>p = 0.289	-0.14<br>p = 0.242	-0.18<br>p = 0.144	0.02<br>p = 0.855
Tree cover	-0.11<br>p = 0.372	-0.09<br>p = 0.486	-0.25<br>p = 0.036*	-0.04<br>p = 0.754
Green space size	-0.06<br>p = 0.644	0.10<br>p = 0.425	-0.10<br>p = 0.398	-0.11<br>p = 0.376

*Significant correlation, given significance level α = 0.05

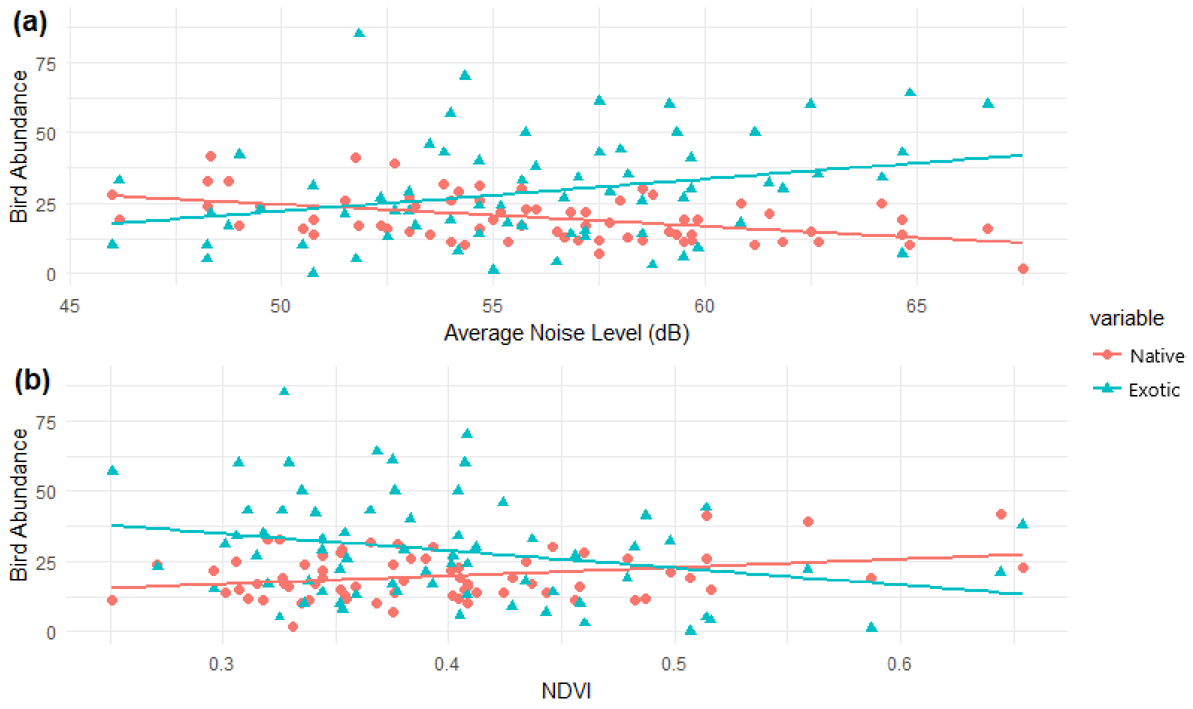

Appendix S4 Relationships between native (red) and exotic (blue) bird abundance and **(a)** average noise levels in green spaces and **(b)** average NDVI.

Appendix S5 Pre-determined detection and occupancy models.

	Model	Covariates
**Detection (p)**	p(.)	Constant detection
	p(MN)	Maximum noise during survey
	p(H)	Average building height surrounding green space
	p(D)	Proportion building cover surrounding green space
	p(NDVI)	Average NDVI in green space
	p(Tree)	Proportion tree cover in green space
	p(NV)	Proportion native vegetation in green space
	p(H + D)	Average building height + Proportion building cover
	p(MN + H)	Maximum noise during survey + Average building height
**Occupancy (ψ)**	ψ(.)	Constant occupancy
	ψ(MNA)	Average maximum noise level in green space during study period
	ψ(H)	Average building height surrounding green space
	ψ(D)	Proportion building cover surrounding green space
	ψ(NDVI)	Average NDVI in green space
	ψ(Tree)	Proportion tree cover in green space
	ψ(NV)	Proportion native vegetation in green space

$\psi(H + D)$	Average building height + Proportion building cover surrounding green space
$\psi(MNA + H)$	Average maximum noise level during study period + Average building height surrounding green space
